# Evanescent Hyperemia: An Underrecognized Cutaneous Manifestation of Postural Orthostatic Tachycardia Syndrome

**DOI:** 10.7759/cureus.105923

**Published:** 2026-03-26

**Authors:** Muhammad Usman Ilyas, Sofia Barlas, Momina Abid, Mohammad Hussain

**Affiliations:** 1 Medicine, Shalamar Medical and Dental College, Lahore, PAK; 2 Osteopathic Medicine, New York Institute of Technology College of Osteopathic Medicine, New York, USA; 3 Medicine, University Medical and Dental College, Lahore, PAK; 4 Internal Medicine, Mayo Clinic, Jacksonville, USA

**Keywords:** evanescent hyperemia, hyperemia, pots, presyncope, prolactinoma

## Abstract

Postural orthostatic tachycardia syndrome (POTS) is characterized by orthostatic tachycardia with associated symptoms including presyncope, fatigue, dizziness, and gastrointestinal complaints, among others. In addition to cardiovascular and neurologic features, autonomic dysfunction may involve other organ systems. We report a rare case of transient evanescent hyperemia occurring during presyncopal episodes in a patient with POTS, highlighting a potentially underrecognized dermatologic sign of autonomic dysfunction.

We present a 19-year-old female with known POTS and a complex medical history including pituitary adenoma (prolactinoma), secondary adrenal insufficiency, gastroparesis, severe malnutrition, and pelvic floor dysfunction, who was admitted for recurrent presyncope, syncope, and collapse. Her presentation was multifactorial. Contributing factors included autonomic instability, cabergoline-related effects, and nutritional compromise. During hospitalization, she developed recurrent episodes of transient, sharply demarcated erythematous patches affecting the face, chest, and upper extremities that coincided with presyncope and resolved spontaneously without intervention. Dermatology evaluation supported a diagnosis of evanescent hyperemia in the setting of autonomic dysfunction associated with POTS. Diagnostic workup included serial laboratory testing, electrocardiography, echocardiography, neuroimaging, and multidisciplinary specialty consultations. Transthoracic echocardiography demonstrated a patent foramen ovale with preserved cardiac function, without evidence of structural heart disease contributing to her symptoms. Management required a multidisciplinary approach, including stress-dose intravenous hydrocortisone for adrenal insufficiency, adjustment of cabergoline due to suspected medication-related bradycardia, continuation of fludrocortisone for volume support, and initiation of nasoduodenal tube feeding for nutritional rehabilitation.

This case illustrates a transient cutaneous finding temporally associated with presyncope in a patient with POTS and complex comorbidities. Awareness of such skin changes during symptomatic episodes may provide supportive clinical clues to underlying autonomic dysfunction, particularly in diagnostically challenging presentations.

## Introduction

Postural orthostatic tachycardia syndrome (POTS) is a form of autonomic dysfunction characterized by an excessive increase in heart rate upon standing, typically a rise of ≥30 bpm (≥40 bpm in adolescents) within 10 minutes of standing or tilt-table testing, accompanied by symptoms of orthostatic intolerance such as dizziness, fatigue, palpitations, and nausea [1]. It is estimated to affect 0.1%-1% of the population in the United States [2]. POTS may also manifest with a range of non-orthostatic symptoms and comorbidities, making recognition and diagnosis challenging. Among its varied presentations are cutaneous manifestations reflecting underlying autonomic and vascular dysregulation, including livedo reticularis, Raynaud’s phenomenon, and cutaneous flushing. A less commonly described feature is evanescent hyperemia, characterized by transient, sharply demarcated areas of erythema that appear and resolve within seconds to minutes during episodes of presyncope or autonomic fluctuation [1,3,4].

Although skin findings in POTS have been documented, with dermatologic abnormalities reported in the majority of patients in some cohorts, evanescent hyperemia remains underrecognized. It was noted as a potentially characteristic sign of hyperadrenergic/low-flow subtypes of POTS, which is characterized by excessive sympathetic activation, whereas the low-flow subtype involves impaired peripheral vasoconstriction and reduced venous return; both mechanisms may predispose to transient cutaneous hyperemia during autonomic fluctuations.

## Case presentation

A 19-year-old female with a known history of POTS, pituitary adenoma (prolactinoma), secondary adrenal insufficiency, gastroparesis, severe malnutrition, and pelvic floor dysfunction presented with recurrent episodes of syncope, presyncope, fatigue, and collapse. This episode differed from her typical POTS flares, presenting with syncope accompanied by prominent gastrointestinal symptoms, including nausea, abdominal pain, and dizziness.

On admission, she was diagnosed with POTS complicated by multifactorial syncope, attributed to autonomic instability, intermittent bradycardia, volume depletion, medication effects, and severe malnutrition. Diagnostic evaluation included serial laboratory testing, electrocardiograms, continuous telemetry monitoring, chest radiography, computed tomography of the head, and transthoracic echocardiography. Echocardiography revealed a patent foramen ovale with left-to-right shunting but otherwise preserved cardiac structure and function. Cardiology and endocrinology consultations concluded that her syncope and bradycardia were most consistent with autonomic dysfunction, medication effects, particularly cabergoline, and nutritional compromise rather than underlying structural cardiac disease.

Given concern for adrenal crisis, the patient was initially treated with stress-dose intravenous hydrocortisone, followed by a gradual taper to her home oral regimen as symptoms and laboratory parameters stabilized. Cabergoline was dose-reduced and intermittently withheld due to suspected medication-induced bradycardia and suppressed prolactin levels. Fludrocortisone therapy was continued as part of volume expansion and dehydration management.

**Figure 1:**
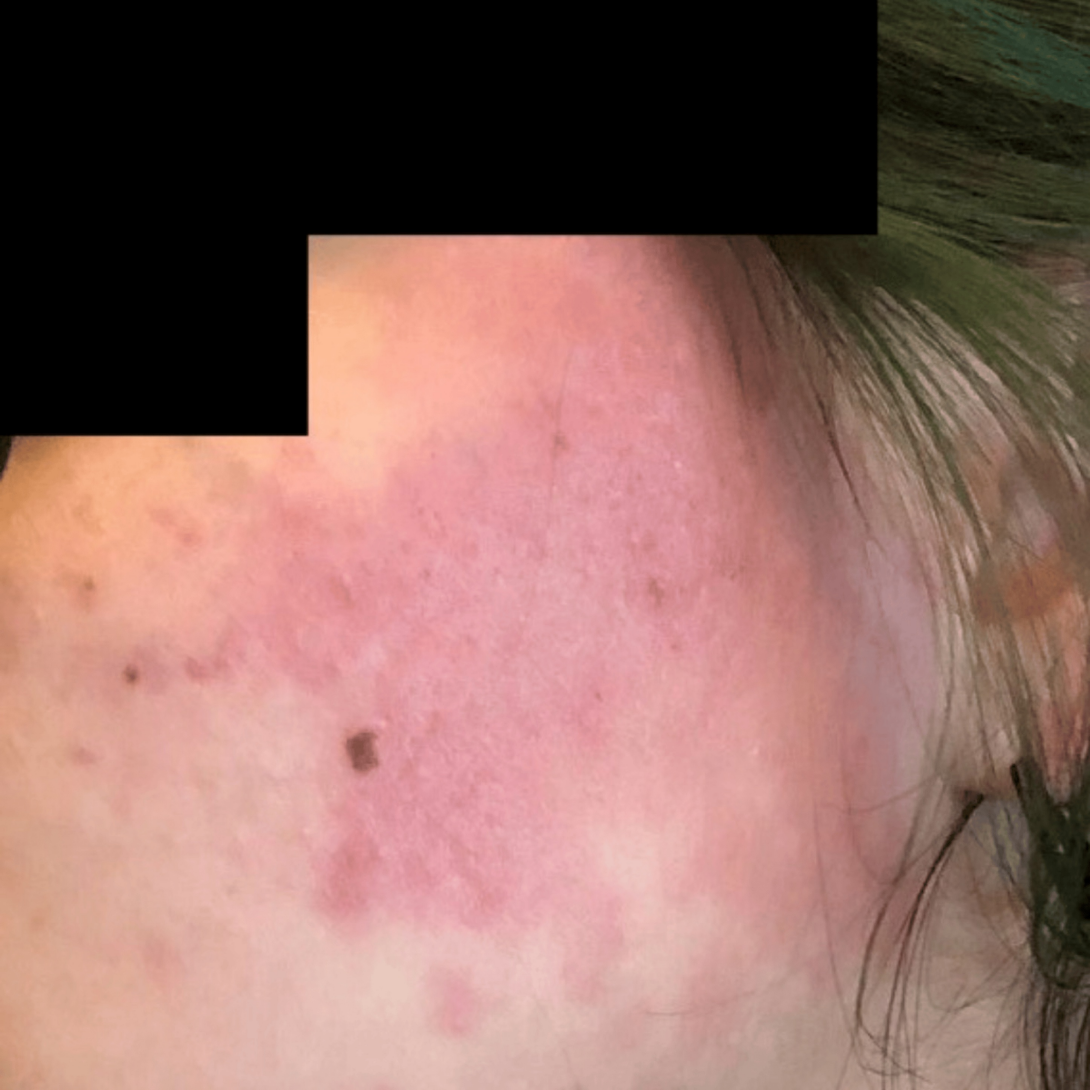
Left side of the face during an episode of evanescent hyperemia

**Figure 2:**
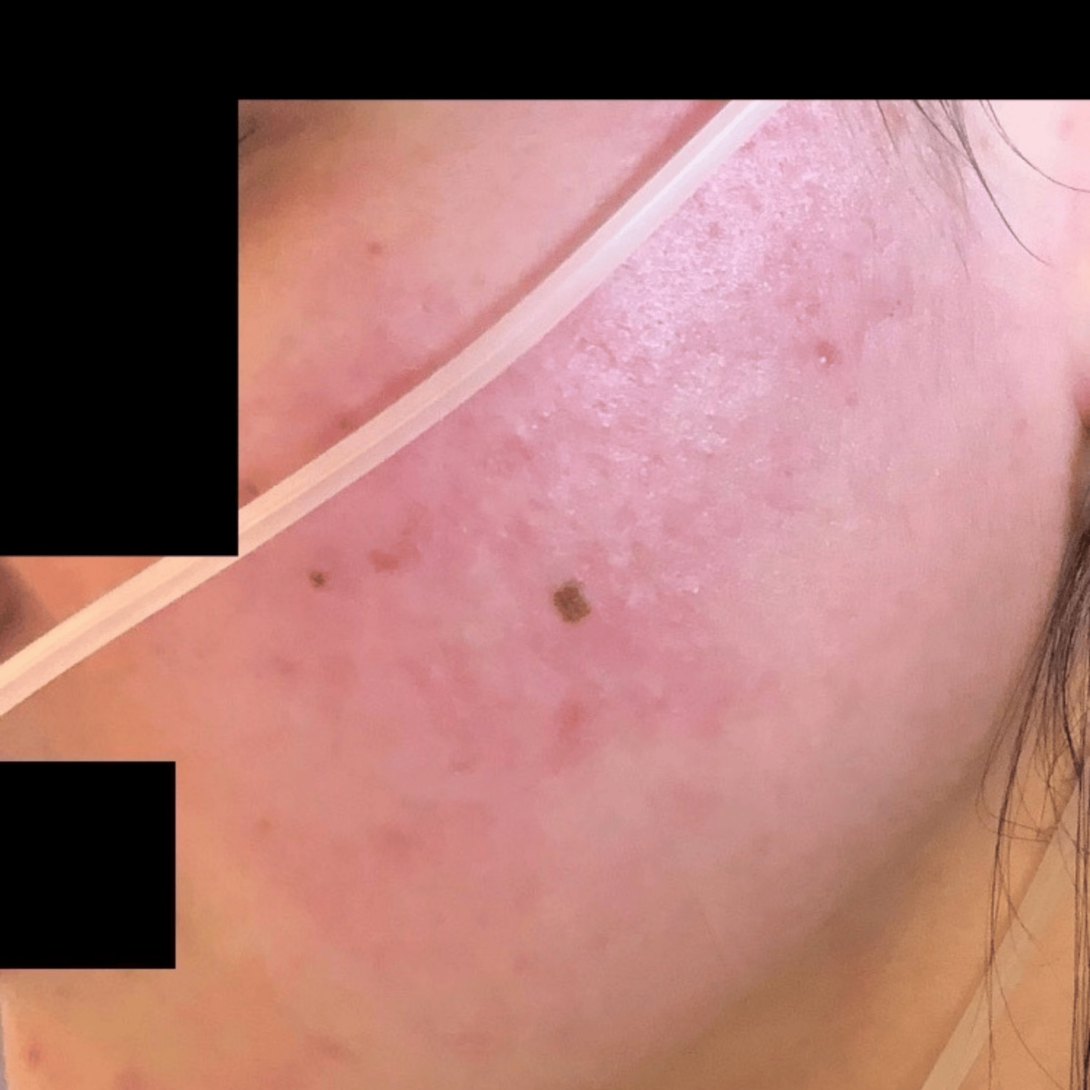
Left side of the face during an episode of evanescent hyperemia

**Figure 3:**
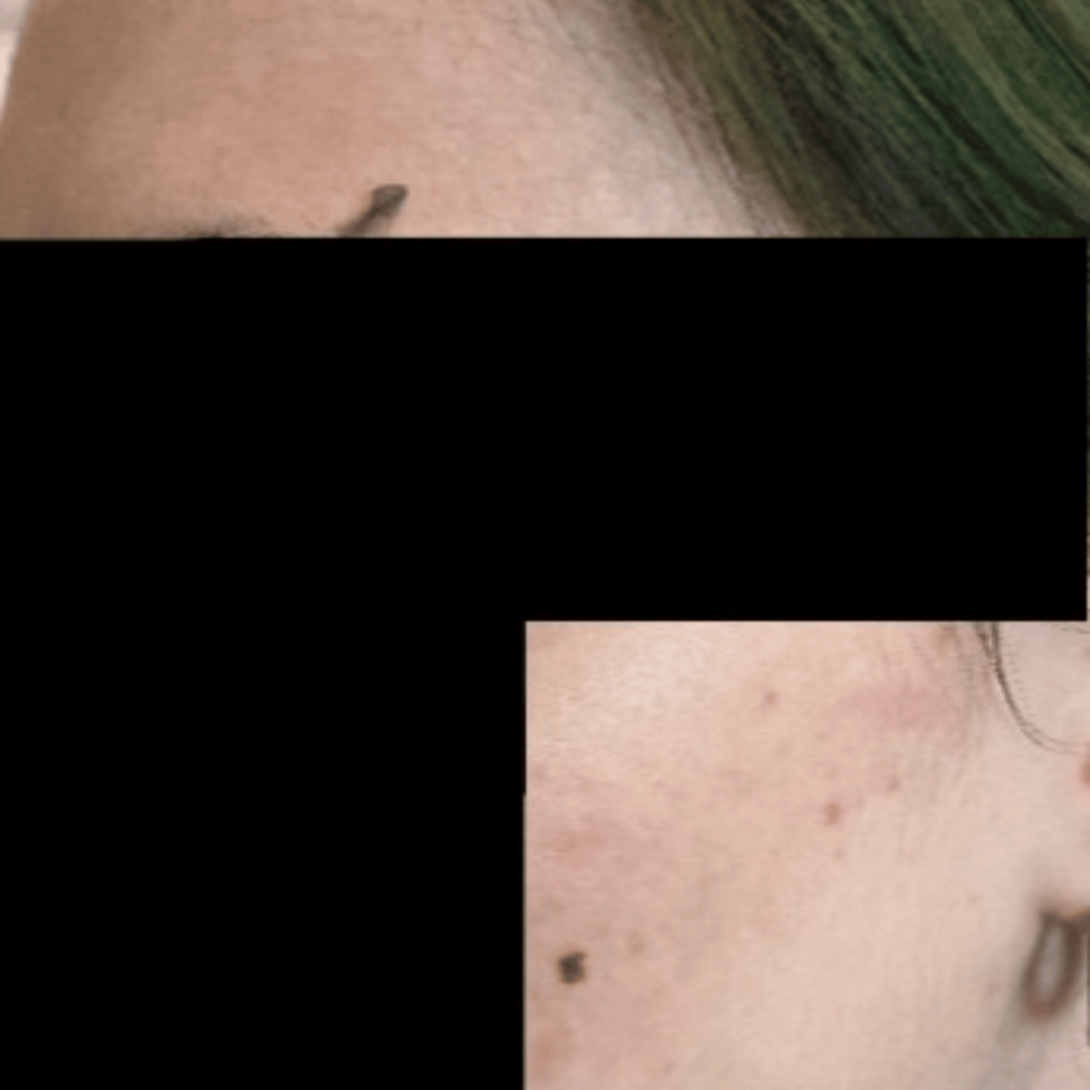
Post-hyperemia left side of the face

**Figure 4:**
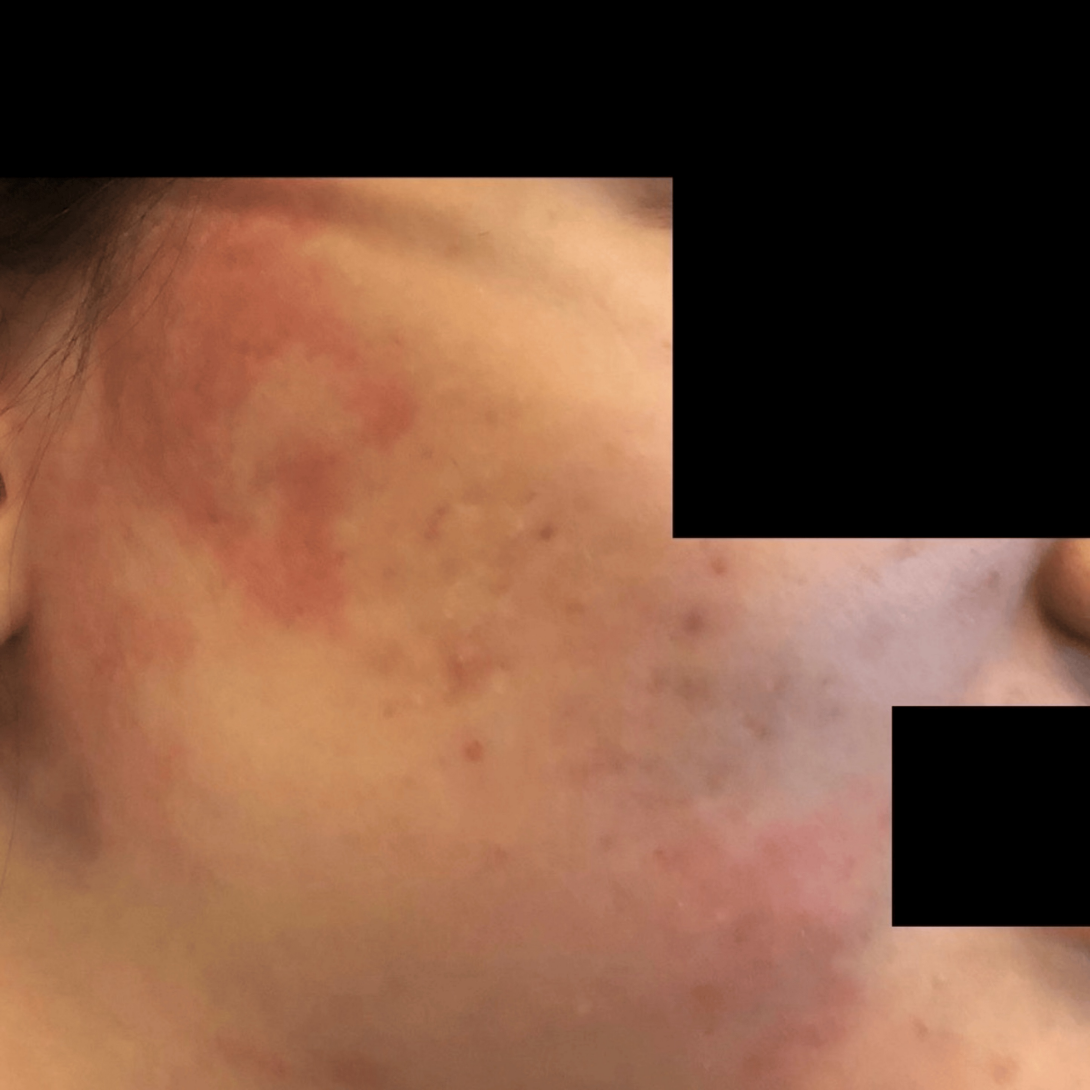
Right side of the face during an episode of evanescent hyperemia

**Figure 5:**
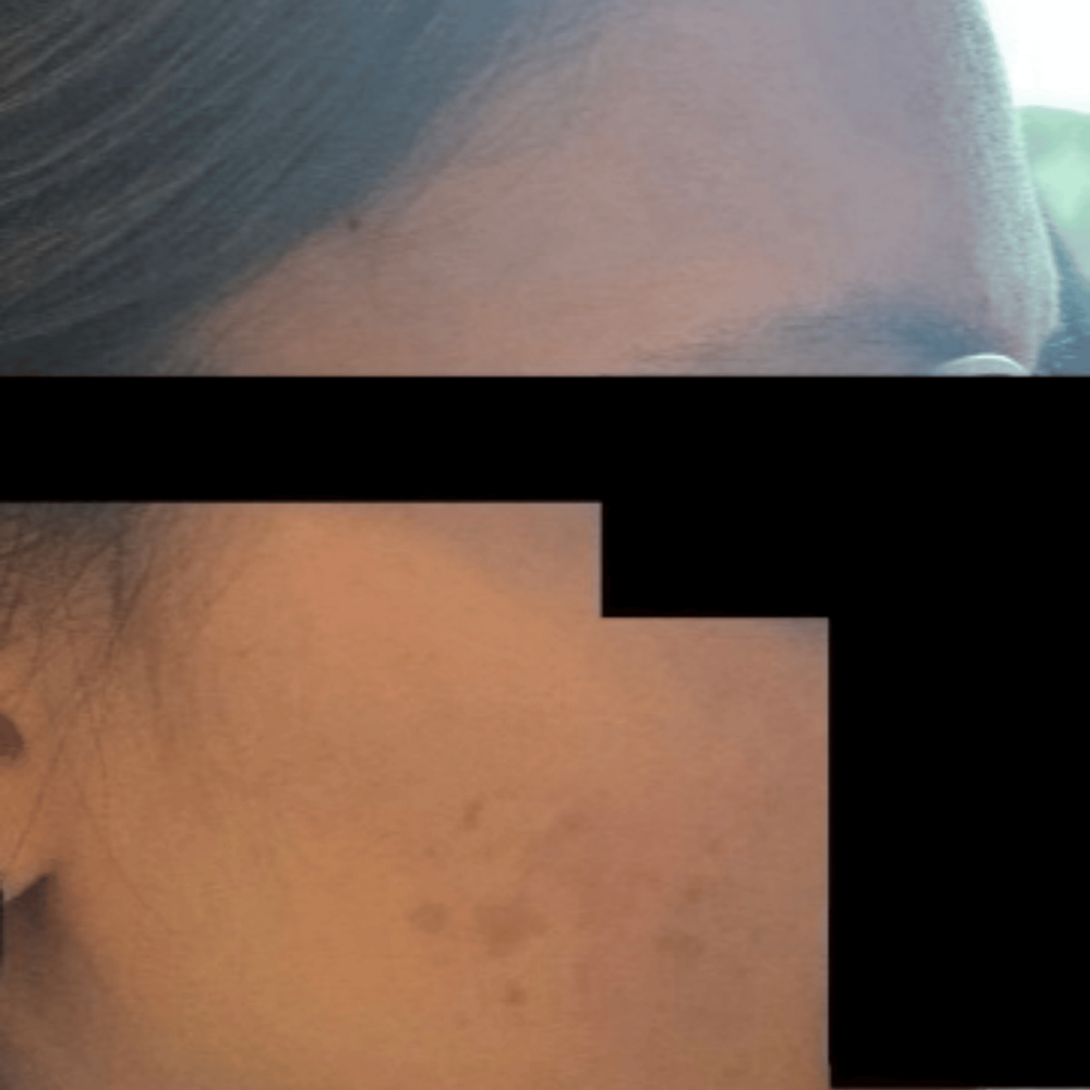
Post-hyperemia right side of the face

Due to poor oral intake in the setting of gastroparesis and severe malnutrition, a nasoduodenal feeding tube (Corpak; Avanos Medical, Alpharetta, GA) was placed. Enteral nutrition was initiated per institutional protocol. The patient was closely monitored for electrolyte disturbances and signs of refeeding syndrome. Over the course of hospitalization, she experienced recurrent presyncopal episodes characterized by postural tachycardia. During these episodes, she developed evanescent hyperemia, manifested as transient, sharply demarcated erythematous patches involving the face, chest, and upper extremities, that appeared for a few minutes and then resolved spontaneously without intervention. Clinical photographs demonstrate the right and left sides of the face before and during episodes of hyperemia, highlighting the abrupt onset and transient nature of the cutaneous changes (Figures 1-5).

Dermatology consultation confirmed these cutaneous findings as consistent with POTS-related autonomic and vascular dysregulation. Physical therapy evaluation revealed significant deconditioning and generalized weakness, prompting recommendations for inpatient rehabilitation and home safety modifications. Constipation related to pelvic floor dysfunction and limited oral intake improved following initiation of enteral feeding and an appropriate bowel regimen. Additional complaints of chest pain and headaches were evaluated and determined to be non-cardiac and non-neurologic in origin. Thyroid function testing demonstrated a non-thyroidal illness pattern, for which no acute intervention was required during this admission.

## Discussion

The dermatologic manifestations of POTS are diverse and include Raynaud’s phenomenon, livedo reticularis, cutaneous flushing, and erythromelalgia [1]. Among these, evanescent hyperemia is a particularly notable but underrecognized finding. It represents transient episodes of cutaneous vasodilation resulting from autonomic and vascular dysregulation and must be carefully differentiated from other causes of flushing. Unlike diffuse or prolonged flushing, evanescent hyperemia demonstrates abrupt onset, sharply demarcated borders, and spontaneous resolution within minutes [1].

Recent patient cohort data suggest that dermatologic manifestations in POTS are highly prevalent, with a large proportion of patients reporting rash and digital color changes, further highlighting the diagnostic relevance of cutaneous signs in autonomic dysfunction [1].

Although characteristic of POTS, evanescent hyperemia is less commonly recognized in clinical practice. Unlike the more persistent signs such as livedo reticularis or Raynaud’s phenomenon, evanescent hyperemia is defined by its sharply demarcated erythematous patches and transient nature, occurring abruptly during orthostatic stress and resolving shortly thereafter, typically within minutes [3,4].

Case reports have emphasized that episodic erythema in POTS is frequently posture-dependent and may be overlooked unless directly observed during symptomatic orthostatic episodes, contributing to underrecognition of this cutaneous manifestation [5]. Additionally, periodic facial erythema has been reported to preferentially involve the cheeks and periorbital regions and is often misattributed to anxiety or primary dermatologic conditions such as rosacea, further delaying recognition of underlying autonomic dysfunction [5].

In the presented case, the patient’s evanescent hyperemia closely correlated with symptomatic orthostatic tachycardia and presyncope, underscoring its clinical relevance as a visible marker of autonomic instability. This patient’s particular case was complex due to the multisystem nature of her disease and the presence of significant comorbidities, necessitating involvement from multiple specialties, including cardiology and endocrinology. Her symptoms of autonomic dysfunction and syncope were likely exacerbated by her secondary adrenal insufficiency, malnutrition, and gastroparesis.

Hypovolemia, whether central or secondary to nutritional compromise, can drive the symptoms of POTS [6]. The chest pain and headache in the patient may be explained by a hyperadrenergic state, further contributing to dysregulation of the autonomic nervous system [6]. Proposed mechanisms include abnormal vascular responsiveness and dysregulation of the renin-angiotensin system, with some reports noting improvement of cutaneous manifestations following angiotensin receptor blockade [3].

With its heterogeneous and multifactorial pathophysiology, management guidelines for POTS emphasize a multidisciplinary and individualized approach, particularly among young adults [6]. Initial management focuses on identifying and addressing reversible contributors, such as discouraging prolonged bed rest and evaluating coexisting chronic conditions. While arrhythmias are commonly treated with radiofrequency ablation therapy, POTS is not primarily an arrhythmic disorder and may not benefit from such interventions [7].

Pharmacologic intervention in POTS patients usually begins with discontinuation of medications that may exacerbate tachycardia or hypovolemia, such as vasodilators, diuretics, and serotonin-norepinephrine reuptake inhibitors (SNRIs). Since many POTS patients are young women, hormonal therapies must also be carefully evaluated; oral contraceptives containing drospirenone may worsen hypovolemia and should be substituted with alternative progestins when appropriate. When hypovolemia is suspected, fludrocortisone, a synthetic mineralocorticoid, is commonly used to expand plasma volume and promote sodium retention, though potential side effects include acne, headache, hypokalemia, and fluid retention. Desmopressin (DDAVP) may provide short-term symptomatic relief by promoting free water retention but carries a risk of hyponatremia. Lastly, midodrine, an α1-agonist vasoconstrictor, may be particularly useful in neuropathic POTS, where impaired vascular tone leads to excessive venous pooling [7].

This report is limited by its focus on a single patient, which restricts generalizability. Observations such as the association of evanescent hyperemia with autonomic symptoms are descriptive and do not establish causality. Larger studies are needed to confirm the prevalence and clinical significance of these findings in patients with POTS.

## Conclusions

Evanescent hyperemia is a distinctive and underrecognized dermatologic manifestation of POTS that can provide a visible clue to underlying autonomic instability, particularly when occurring in a posture-dependent and transient pattern. Recognition of this rash, along with careful assessment of coexisting conditions such as adrenal insufficiency, malnutrition, and gastrointestinal dysmotility, can improve timely diagnosis and guide individualized management. This case underscores the importance of multidisciplinary evaluation in complex cases and highlights the diagnostic and clinical relevance of cutaneous signs of POTS. Greater awareness of evanescent hyperemia may prevent misdiagnosis, reduce delays in treatment, and enhance patient outcomes.
